# Recall of Briefly Presented Chess Positions and Its Relation to Chess Skill

**DOI:** 10.1371/journal.pone.0118756

**Published:** 2015-03-16

**Authors:** Yanfei Gong, K. Anders Ericsson, Jerad H. Moxley

**Affiliations:** 1 School of Psychology and Cognitive Science, East China Normal University, Shanghai, China; 2 Department of Psychology, Florida State University, Tallahassee, Florida, United States of America; VU University Amsterdam, NETHERLANDS

## Abstract

Individual differences in memory performance in a domain of expertise have traditionally been accounted for by previously acquired chunks of knowledge and patterns. These accounts have been examined experimentally mainly in chess. The role of chunks (clusters of chess pieces recalled in rapid succession during recall of chess positions) and their relations to chess skill are, however, under debate. By introducing an independent chunk-identification technique, namely repeated-recall technique, this study identified individual chunks for particular chess players. The study not only tested chess players with increasing chess expertise, but also tested non-chess players who should not have previously acquired any chess related chunks in memory. For recall of game positions significant differences between players and non-players were found in virtually all the characteristics of chunks recalled. Size of the largest chunks also correlates with chess skill within the group of rated chess players. Further research will help us understand how these memory encodings can explain large differences in chess skill.

## Introduction

In his classic paper, Miller [1] proposed that limited storage capacity of human short term memory (STM) [2], could be expressed as seven plus/minus two chunks, where each chunk corresponded to a familiar pattern previously stored in long-term memory (LTM). Simon and Chase [3, 4] extended chunking theory to account for large individual differences between expert and novice chess players in their recall of chess positions. They proposed that experts did not differ from novices in their memory capacity but that the experts’ superior recall for game positions was due to the recall of larger chunks involving a larger number of chess pieces. The experts’ large recall advantage did not transfer to randomly rearranged pieces in positions because the meaningful structure of the chess position was destroyed by the randomization, thus essentially eliminating opportunities for more skilled players to perceive familiar chunks. Consistent with their theoretical predictions experts in many other domains showed a recall advantage over less accomplished performers in the same domains, but only for representative structured stimuli (see [5, 6] for review).

The concept of chunking is central to many cognitive theories (Soar, [7]; Template Theory, [8]) but the majority of research on the characteristics of chunks has been collected in the domain of chess. Recently the role of perceptual patterns (chunks) and abstract semantic relations mediating the encoding of chess positions for players at different levels of chess skill is being debated [9, 10]. To address some of these issues our study examines the nature of chunks and their relation to chess skill by re-examining their structure and characteristics for a wide range of chess skill by including individuals, who do not play chess.

In the Chase-Simon model of expertise [3, 4] and its extension [8], a chunk is represented as a combination of a series of location-specific chess pieces [11, 12, 13]. This assumption has been viewed as inconsistent with experts’ encodings of the gist of a chess position [14] that would be required for drawing analogies between different chess positions [15, 16]. The location-specific characteristics of chunks have been supported by a series of studies where players attempted to recall briefly presented dramatically modified chess positions [17, 18]. On the other hand evidence for abstract relations was reported by Linhares and Brum [15], who found that more skilled chess players were more able to categorize chess positions based on abstract relations than less skilled players. In their response Bilalić and Gobet [19] argued that Linhares and Brum’s participants categorized positions based on abstract relations because they had been instructed to do so, and that chess positions could be categorized by concrete or abstract similarity depending on the instruction. In their reply Linhares and Brum [20] argued that experts were able to encode positions at an abstract level whereas novices were not able to do so. In a recent commentary Charness [21] proposed that skilled chess players need a wide range of representations and that they are likely to use different representations depending on the requirements of the tasks. To assess similarities and differences in the representation used to complete the different chess-related tasks as a function of chess skill we must be able to identify the representations used in each task.

In their pioneering work, Chase and Simon [4] showed that relations between chess pieces recalled in sequence within a chunk differed systematically from the relations of chess pieces recalled in sequence but belonging to different chunks—a major source of evidence validating the existence of chunks. When Linhares and Freitas [9] restricted their analysis to relations of chess pieces within chunks they did not find significant differences in the relative pattern of chess relations for players at different levels of chess skill. With two cycles of presentation and reconstruction, differences in the type of recall errors, in particular missed attacks, differentiated players with high and low skill [22]. Lane and Gobet [10] agreed that the correlations between patterns of chess relations within chunks did not differentiate between players differing in skill. In fact, they concluded that all chess players used “the same perceptual building blocks” for recalling chunks.

In order to test if non-chess players have similar perceptual building blocks as chess players we will compare a full sample of non-chess players, who would be predicted to have acquired no chunks and templates, with a skilled sample. We decided to use the term *chunk* as an operationalization to indicate groupings of chess pieces identified in the recall of any participant, thus including non-chess players lacking specialized knowledge about chess playing.

Our study largely replicates the Chase-Simon [4] study of chess memory for game and random positions by using their multi-trial free recall paradigm. Compared to other studies in chess which employed the multi-trial free recall paradigm [22, 23, 24], we use computer-based presentation for 5 s and recording of recall responses [25]. Our samples of participants are considerably larger, that is participants were recruited along the continuum of age both in the skilled sample and the non-chess sample. We also use a larger sample of representative game and random stimuli. In addition to identifying chunks with the traditional RT technique [4, 25] we introduce a refined method, namely the repeated-recall technique. By comparing these two completely independent techniques we can assess the reliability and validity of the chunk hypotheses during recall and assess whether one of the techniques is superior to the other.

The repeated-recall technique is an adaptation of the ordered tree technique [26]. In this method we identified a group of pieces as a chunk if they were recalled together, without any other pieces inserted, on two memory trials. The order of pieces recalled on the first trial was compared to those on the second trial, as well as to those on the last trial to make the estimations of chunks more conservative. Although Chase and Simon’s classic RT technique has been applied to identify chunks for samples of players who differed in age and/or skill level [4, 13, 27], it failed to identify chunks for the recall of a GO master, whose chunks were neither organized in a strictly nested way nor in a linear way [28, 29]. Slower response times for old participants even among chess experts doing simple chess tasks [30] creates potential problems for the RT-technique with fixed criterion for pause length. In contrast, the repeated-recall technique addresses most of these criticisms raised against the RT technique. The time taken to recall chess pieces is not germane to the identification of chunks and thus makes this technique particularly valuable for memory studies with older participants, whose speed of performance is slower than younger participants [31].

The major computational model for describing recall of game and random chess positions (CHREST) has made quantitative predictions about relations between chunk characteristics and chess skill [13, 32]. These predictions include a reduced (but still present) expertise advantage in random positions. Stronger players recall more pieces and larger largest chunks on game positions. CHREST predicts essentially no difference in number of chunks recalled in very brief presentations for both game and random boards. Finally CHREST predicts that within-chunk pieces will have more chess relationships than between-chunk pieces. We re-examined how characteristics of chunks identified for non-chess players and chess players might differ using the repeated-recall technique for identifying chunks and how they relate to chess skill and memory for chess positions.

## Methods

### Ethics statement

This study received approval from the Florida State University Human Subjects Committee (HSC No. 2011.5687 ICF 1–3). All participants read and signed the written informed consent prior to their participation. Participants under 18 had to be accompanied by their parents who read and signed the parental consent form, and the minors also signed an assent form.

### Participants

After testing 60 individuals, 25 players with chess ratings were identified and assigned to the group of chess players. From the same pool 25 individuals were identified and assigned to the group of non-chess players based on their lack of chess knowledge, operationalized by their failure to accurately report the moves of a knight or a pawn. Consequently, 10 non-rated chess players with more chess knowledge and experience in chess playing were excluded from further analyses.

The 25 rated chess players were recruited from chess clubs in Florida. Their skill levels ranged from class H to Expert (see Table 1). Non-rated chess players were recruited by advertising in a manner to match the age and years of education of the rated chess player group. The biographic information of participants is shown in Table 1. Rated chess players did not differ from non-chess players in age, *F* (1, 48) = 1.27, *ns*, nor in years of education, *F* (1, 48) = 0.06, *ns*. Participating college students in psychology classes were compensated with credits toward course requirements and other participants were paid on an hourly basis.

**Table 1:** Participants’ biographic information.

Skill Group	Descriptives	USCF Rating ^a^	Age	Years of education
Rated chess players (N = 25)	Range	(411, 2166)	(8, 80)	(3, 22)
	Mean	1679.75	38.08	15.48
	SD	434.70	20.73	4.81
Non-chess players (N = 25)	Range		(10, 74)	(4, 29)
	Mean		31.56	15.24
	SD		20.22	5.09

^a^ The United States Chess Federation (USCF) rating system, proposed by Mark Glickman, was transitioned from the Elo rating system.

The skill levels are similar in those two systems: chess players with more than 2200 points are called masters (from 2200 to 2399 are national masters, from 2400 and up are senior masters), from 2000 to 2199 are experts, from 1800 to 1999 are class A, etc. The ratings used by different organizations, such as FIDE and USCF are not always directly comparable. USCF ratings are generally 50 to 100 points higher than the FIDE equivalents. For example, someone with a FIDE rating of 2500 will generally have a USCF rating near 2600.

### Materials

Eight middle game positions were selected from grandmasters’ games in chess tournaments, where the chess position had white to move after 20 moves (40 plies). All eight game positions had between 22 and 28 pieces. The game positions were matched in pairs so there would be a similar number of pieces on the board and from each pair we selected one position randomly, and randomized the positions of the chess pieces by using a randomizing function in MATLAB.

A 14-inch pure-blue screen on a laptop was used to present the chess positions. The positions were presented with a 12 ×12 cm chessboard with each square being 15 ×15 mm. Shapes of pieces were chess standard. On the top of the screen there were buttons, which were used to start the trials and save the final results. There was also a “piece area” below the display of the chessboard, where the 6 different chess pieces of each color were arranged (with king, queen, rook, bishop, knight, pawn into two rows—one for each color with the white pieces as the upper row). Each piece was the same size as each square. Once a piece was selected, a red square around it appeared. A black screen was shown for 2 s between each presentation and each reconstruction. Participants needed to select a piece from the “piece area” and release the piece at its destination square. Each action was performed with a mouse click.

### Procedure and design

In the main chess memory task, there was a block of 5 Trials to present and recall each chess position (Board). The presentations of chess positions were counterbalanced for two presentation times (1 or 5 s) and the type of position (game or random). This led to the presentation of two different boards (Board 1 and Board 2) for each of the four experimental conditions, namely game positions presented at 5 s and at 1 s and random positions presented at 5 s and at 1 s. Other major studies of memory for chess positions presented only 2 or 3 different positions per experimental condition [13, 33]. Participants were asked to try to memorize as many pieces as they could when the positions were presented to them on the computer screen. Following the presentation they recalled the position on a blank board displayed on the computer screen. The participants were instructed to recall as fast as possible while maintaining accuracy and thus refrain from guessing. Participants were informed of the type of chess position (game or random) and the presentation time (1 or 5 s) in advance of the start of each Trial. Before the start of the main experiment, participants received 2 warm-up positions (different from the positions used in the main experiment) with one game position presented for 1 s and one random position presented for 5 s. To save time, each of these 2 positions was presented for 3 cycles of presentation-recall (Trial). Most previous research has focused on memory performance in the 5 s condition so this paper will only analyze data from that condition.

### Identifying chunks by the repeated-recall technique

**Fig 1:**
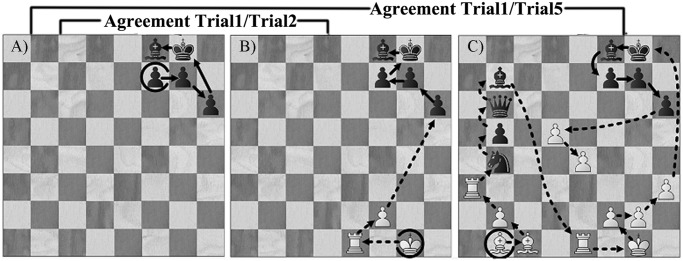
An example of how to identify chunks by comparing order of pieces recalled in Trial 1 (A) to Trial 2 (B), and comparing Trial 1 to Trial 5 (C), the open-circled piece indicates the start of recall.

Our study focused primarily on the first recall of a chess position on Trial 1 made immediately after the first presentation of a given position. The order of recall of pieces on Trial 1 was compared to the order of recall of pieces on Trial 2, referred to as Agreement_Trial1/Trial2_, as well as compared to the final recall of pieces on Trial 5, referred to as Agreement_Trial1/Trial5_ (see Fig. 1). All pieces recalled together in any order on both recall trials were recognized as a chunk as long as there were no other pieces inserted between them. In sum, our technique identified chunks in which several pieces are recalled together as well as a single piece recalled in the same location on both trials, namely single-piece chunks, given that they are the basic units of perception of chess positions [28] and that they are valid especially for non-chess players.

Gobet and Simon [25] considered all pieces, both correct and incorrect, when they identified sequences of chess pieces recalled without a pause (chunks). They argued that incorrect pieces share the same psychological meaning as correct pieces, since they both may arise from chunks stored in LTM. In contrast, Charness [34] restricted his analysis to correctly recalled pieces, which he referred to as “correct chunks”. In our analysis, incorrectly recalled pieces can be taken as (parts of) chunks, but only if they were reproduced consistently on the two recall trials analyzed with the repeated-recall technique.

### Statistical methods for the analyses

*Linear mixed models (LMM)* were mainly used in the analyses, where the model intercepts and participants were allowed to be random effects. Continuous variables, such as age and USCF rating, were centered in the modeling, and degrees of freedom were generated based on the Laplace procedure. Additionally, *hierarchical multiple regression analysis* was employed to see if additional variance can be explained when certain variables were controlled (c.f. [35]). *Johnson-Neyman regions of significance* [36] was adopted to illustrate the significant interactions involving one continuous variable and one categorical variable and *Bonferroni corrected post-hoc t tests* were used to explore any significant differences in main effects or interactions for categorical variables.

## Results

The first section compares chunks identified with the traditional RT technique with those identified with our new repeated-recall technique in terms of their reliability, validity and ability to account for overall memory performance. The second section analyzes chunks identified with the repeated-recall technique and tests for differences in the two traditional characteristics of chunks, namely maximal size and number of different chunks, as a function of chess knowledge (chess players versus non-chess players), type of position (random vs game) and age. In the third section we present the analysis of two additional chunk characteristics measuring chess- and skill-related attributes, such as semantic relations between within-chunk pieces and recall order consistency for within-chunk pieces. The fourth section examines chunk characteristics’ relations to overall memory performance for game positions and chess skill.

### Comparison of techniques for assessing chunks during recall

Following Chase and Simon’s [4] analyses we identified groups of chess pieces (chunks) that were recalled together during the recall with the repeated-recall technique and the traditional RT technique.

**Chunks identified by the repeated-recall technique.** To test the consistency of our measures of identifying chunks, namely Agreement_Trial1/Trial2_ and Agreement_Trial1/Trial5_, we then calculated the number of pieces that were part of chunks on Trial 2 to see how many of them were part of chunks identified on Trial 5. We found that 93% of the chunks on Trial 1, when compared to Trial 2 (Agreement_Trial1/Trial2_), were also identified for the recall on Trial 5 (Agreement_Trial1/Trial5_), and in 84% of the analyzed comparisons the correspondence was perfect (100%). The identification of chunks with the two measures, allowed us to calculate two independent estimates of number of chunks and the largest chunk for the recall on Trial 1 for every presented chess position. To assess the generalizability of the measures across different chess positions we capitalized on the fact that we presented two positions (Board 1 and Board 2) for each experimental condition (game position and random position). The test-retest reliabilities (Board 1 and Board 2) for largest chunk and number of chunks were all significant for each experimental condition (see Table 2). Given the high agreement of the chunk estimates based on the two measures, we will only report on the Agreement_Trial1/Trial2_ characteristics for chunks (based on the comparison of Trial 1 and Trial 2) in the remaining analyses of this article.

**Table 2:** Correlations between two measures of chunk characteristics for each board under each experimental condition (***N*** = 50).

	Number of chunks		Size of the largest chunk	
Position type	Board 1	Board 2	Board 1	Board 2
Game position	.86***	.80***	.82***	.85***
Random position	.44**	.57***	.74***	.86***

** *p* < .01.

*** *p* < .001.

**Chunks identified by RT technique.** For the same dataset we also identified chunks using the classic RT technique proposed by Chase and Simon [4] during the recall of chess positions immediately after the first presentation of a given position on Trial 1. The associated distribution of inter-piece latencies was positively skewed (7.22), with the majority of latencies being shorter than 2 s and a long tail with longer latencies. The peak (mode) of the distribution of latencies was located around 1 s. This pattern matches the characteristics of the distributions reported by Chase and Simon [4] and Gobet and Simon [25]. Following the recommendations by Gobet and Simon [25], we corrected the observed latencies for the predicted movement times to place individual pieces based on Fitts’ law ([37], pp. 241–242) and then used their criterion of 2 s for distinguishing between-chunk latencies from within-chunk latencies. For each participant’s recall of every position the number of chunks and size of the largest chunk per position were recorded for the first presentation of new game and random positions.

The RT techniques used by Gobet and Simon [25] and by Chase and Simon [4] differ from each other in some aspects. Gobet and Simon [25] analyzed all recalled chess pieces, regardless of whether they were incorrect or correct, when segmenting chunks. In contrast, in their original study, Chase and Simon [4] excluded pieces which had been recalled after a very long pause (10 s or more). Both of these two techniques included chunks consisting of a single chess piece. When we analyzed the characteristics of chunks identified with the techniques used by Chase and Simon [4] and by Gobet and Simon [25], we found they gave very similar results for our data. The correlations between the estimates of the two techniques for numbers of chunks as well as for sizes of the largest chunks on each of the two boards presented in each of the 2 experimental conditions were 0.85 or higher, *p*s < .001. In the following analyses we will report the data on number of chunks and size of the largest chunks identified with the technique used by Gobet and Simon [25].

**Fig 2:**
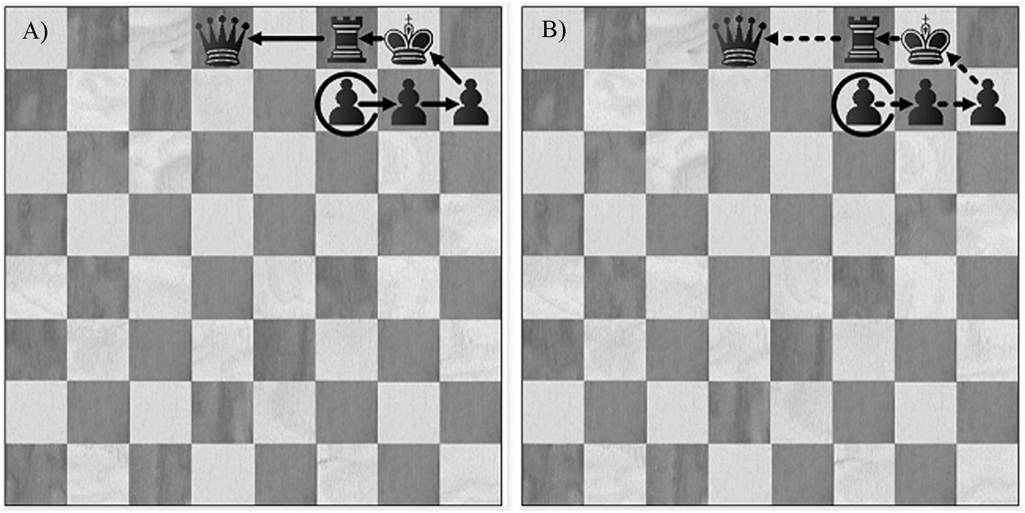
An example of a chunk identified by repeated-recall technique and RT technique, the open-circled piece indicates the start of recall: Arrows with full lines indicate the order of pieces recalled on Trial 1 (A), and arrows with dotted lines indicate the order of pieces recalled on Trial 2 (B).

**A comparison between the two techniques for identifying chunks.** The two different techniques can identify different chunks as illustrated for a given recall of Board 1 in the game position condition. Based on the repeated-recall technique a participant recalled six pieces together on both Trial 1 and Trial 2 (see Fig. 2), which corresponds to a single chunk with six pieces. Based on the RT technique we identified 3 chunks with the largest chunk size of four pieces because the times for placing the black rook and king were longer than 2 s. During the recall of the second game position (Board 2) the same participant recalled 4 black pawns in exactly the same order on Trial 1 and Trial 2. Both techniques identified the same chunk with 4 pieces, because each pawn was placed on the board within 2 s of the preceding piece. The test-retest correlations of chunk characteristics for recall of Board 1 with the chunk characteristics for recall of Board 2 for the two techniques are given separately in Table 3.

**Table 3:** Correlations between Board 1 and Board 2 on characteristics of chunks estimated by two techniques.

		Repeated-recall technique		RT technique	
Participant	Position type	Number	Size	Number	Size
All participants	Game position	.65***	.38**	.36**	.39**
	Random position	.21	.45**	.17	.32*
Rated chess players	Game position	.60**	.29	.36**	.20
	Random position	.14	.12	.14	.13

* *p* < .05.

** *p* < .01.

*** *p* < .001.

When we tested the differences in test-retest reliability for the two techniques based on the data from Board 1 and Board 2, we found that the only statistically significant differences in reliability concerned the game positions (.65 vs .36) with the repeated recall technique producing more reliable estimates for number of chunks [38]. When the analysis was restricted to rated chess players, this estimate of reliability is no longer significantly different for the two techniques for game positions (.60 vs .36).

In a more direct test of the validity of the measures of chunk characteristics we tried to predict the number of pieces correctly recalled for one board (Board 2) based on the chunk characteristics found for the other board (Board 1) presented under the same experimental condition. This procedure avoids the problem of correlating chunk characteristics and number of recalled pieces for the same trial. In addition, CHREST makes a prediction that more skilled chess players recall larger chunks. By this procedure, we might not be able to make an exact estimation of how many chunks players recalled and how many pieces the largest chunks contained on each given position, but we can assess this prediction by summarizing some systematic relations between chunk characteristics and chess skill. The results from the regression analyses are reported in Table 4. The two chunk estimates based on the RT technique were first entered and then, the two chunk estimates based on the repeated-recall technique were entered to assess if additional significant variance in recall was accounted for by the repeated-recall technique. Finally the variables with chunk estimates of the RT technique were removed from the regression equation to assess if any significant contribution was made by the RT estimates beyond the estimates from the repeated-recall technique. The pattern of results is consistent across both experimental conditions, namely the RT technique significantly accounts for the recall performance, and the repeated-recall technique accounts for significant additional variance, but when the estimates of the RT technique are removed there is no significant reduction in accounted variance. In sum, the estimates based on repeated-recall techniques can account for significantly larger portions of variance in recall performance above and beyond the estimates based on the RT technique.

**Table 4:** Hierarchical regression analyses of characteristics of repeated-recall and RT techniques on one Board to predict memory performance on the other Board in each experimental condition.

		After entry of RT chunks		After entry of Repeated-recall chunks		After removal of RT chunks	
Participant	Position type	R^2^	F_change_	R^2^	F_change_	R^2^	F_change_
All participants	Game position	.15	4.20*	.62	27.94***	.61	0.87
	Random position	.14	3.54**	.25	3.12*	.22	0.88
Rated chess players	Game position	.17	3.30	.63	17.56***	.57	2.18
	Random position	.12	1.96	.23	1.97	.19	0.56

* *p* < .05.

** *p* < .01.

*** *p* < .001.

When restricting the same analysis to rated chess players (see Table 4), we found the same pattern for game positions, where RT chunks explained significant variance in the first step and repeated-recall chunks explained significant additional variance, but there was no significant reduction in explained variance when variables measuring RT chunks were removed. Neither type of chunk variables was able to significantly account for memory performance for random positions, all *r* (23)s < .39, *ns*. In sum, the repeated-recall technique identified the characteristics of chunks that were more reliable across memory trials and predicted memory performance significantly better than those identified with the RT technique.

### Do traditional characteristics of repeated-recall chunks relate to chess skill?

Focusing on the repeated recall chunks, we averaged results across Board 1 and Board 2 in each experimental condition for the two basic chunk characteristics, namely number of chunks and size of the largest chunks. It is well established that superior chess skill is associated with better memory performance for game and random positions (e.g., [13, 18, 27, 33, 34, 39]) so we used the USCF rating as the indicator of chess skill and analyzed its relation to chunk characteristics for rated chess players separately after the main analysis for all participants.

**Fig 3:**
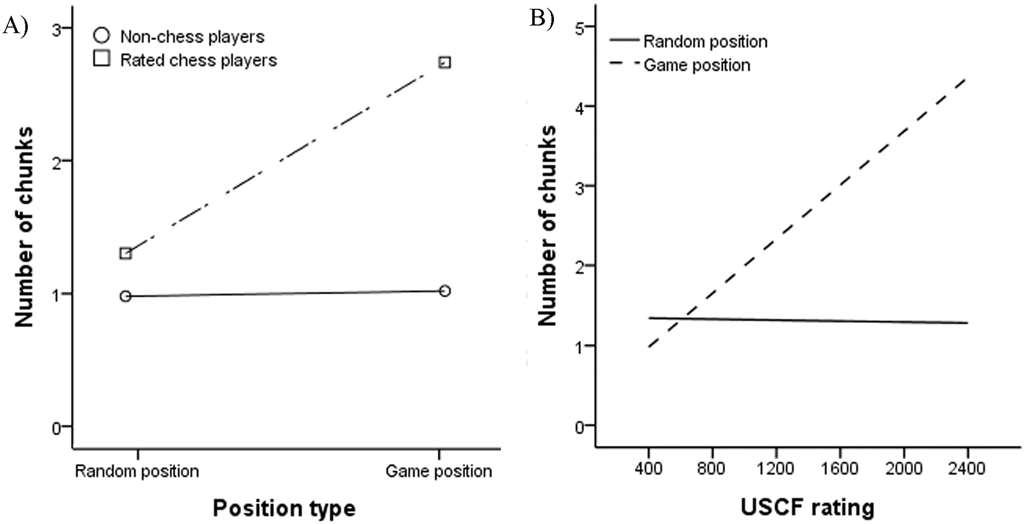
Number of chunks as a function of type of position for different skill groups (A) and number of chunks as a function of chess skill for different types of positions for rated chess players (B).

**An analysis of number of chunks.** An LMM analysis was conducted to analyze number of chunks (see Table 5 for results of the analysis). The main effects of position type and skill group were significant but were qualified by the significant interaction between them (see Fig. 3, A). A post hoc analysis using Bonferroni-corrected *t* tests for the significant interaction showed that 3 out of 6 pair-wise comparisons reached significance (all *t* (48)s > 3.88, *p*s < .001/6), with rated chess players recalling more chunks for game positions (*M* = 2.74, *SD* = 2.08) than they recalled for random positions (*M* = 1.30, *SD* = 0.50) and the non-chess players recalled for game (*M* = 1.02, *SD* = 0.55) and random positions (*M* = 0.98, *SD* = 0.44). Therefore, rated chess players recalled more chunks for game than for random positions, whereas there was no difference for non-chess players.

**Table 5:** The results of linear mixed model analyses for memory performance and two basic chunk characteristics for all players.

	Number of chunks		Size of the largest chunk	
Effect	Df (N, D)	F	Df (N, D)	F
Age	(1, 46)	0.11	(1, 46)	7.28*
Position type	(1, 48)	12.17***	(1, 48)	23.64***
Skill group	(1, 46)	16.54***	(1, 46)	22.15***
Age by skill group	(1, 46)	3.27	(1, 46)	3.98
Skill group by position type	(1, 48)	13.60***	(1, 48)	7.78**

* *p* < .05.

** *p* < .01.

*** *p* < .001.

For rated chess players (see Table 6 for results of the analysis), the only significant main effect of position type was qualified by the significant interaction between position type and USCF rating (see Fig. 3, B). More highly rated chess players recalled more chunks than less rated players for game positions, but there was no significant relation for random positions. USCF ratings did not significantly correlate with the number of chunks for either type of position (see Table 7).

**Table 6:** The results of linear mixed model analyses for memory performance and two basic chunk characteristics for rated chess players.

	Number of chunks		Size of the largest chunk	
Effect	Df (N, D)	F	Df (N, D)	F
Age	(1, 22)	0.06	(1, 22)	4.54*
Position type	(1, 23)	24.62***	(1, 23)	23.28***
USCF rating	(1, 22)	3.9	(1, 22)	8.91**
Position type by USCF rating	(1, 23)	9.10**	(1, 23)	2.98

* *p* < .05.

** *p* < .01.

*** *p* < .001.

**Table 7:** Summary of differences between rated chess players and non-chess players and their relations to chess skill and memory performance.

		Correlations for rated chess players	
	Difference between chess and non-chess players	Memory performance ^a^	Chess skill (USCF rating) ^b^
Number of chunks (Game)	2.74 vs. 1.02 ***	.70***	.29
Number of chunks (Random)	1.30 vs. 0.98	.29	.25
Size of the largest chunks (Game)	4.56 vs. 2.60 ***	.38	.60**
Size of the largest chunks (Random)	2.64 vs. 2.08	.09	.15
Chess relation (Game)	2.81 vs. 2.66	.49*	.05
Semantic relation (Game)	0.47 vs. 0.23 ***	.25	.20
Percentage of different ordered chunks (Game)	0.82 vs. 0.33 ***	.06	.24

* *p* < .05.

** *p* < .01.

*** *p* < .001.

^a^ Memory performance on Board 2 was employed to calculate its correlation with number of chunks and size of the largest chunks for game positions.

Average memory performance for game positions was used to calculate all other correlations.

^b^ Age was controlled when calculating the correlations in the column of “Chess skill (USCF rating)”.

**Fig 4:**
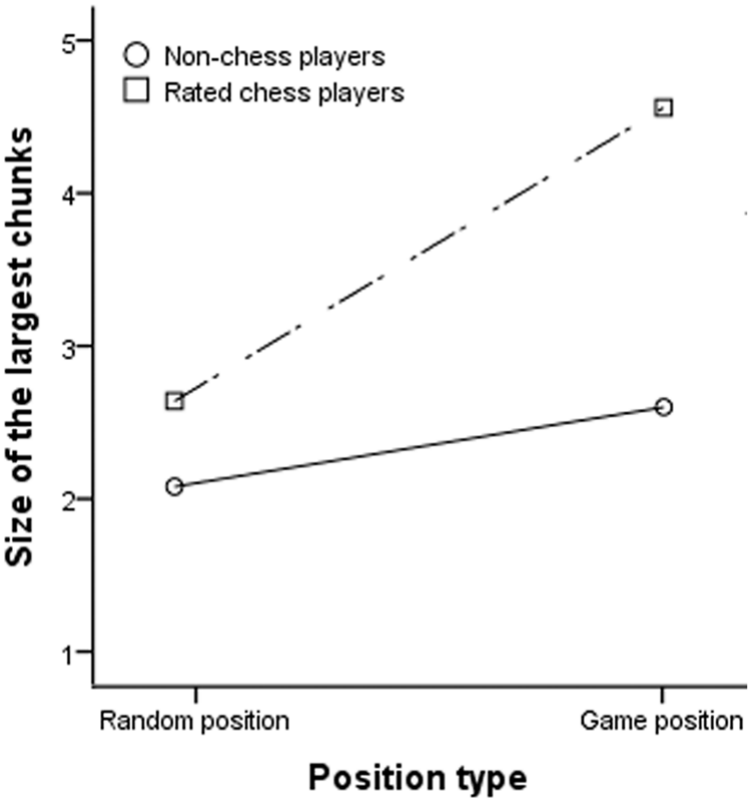
Size of the largest chunks as a function of type of position for different skill groups.

**An analysis of size of the largest chunks.** The same LMM analysis was conducted for the size of the largest chunks (see Table 5 for results of the analysis). Similarly, the main effects of position type and skill group were significant but were qualified by the significant interaction between them (see Fig. 4). Bonferroni-corrected post-hoc analyses using *t* tests for the interaction showed that 3 out of 6 pair-wise comparisons were significant (all *t* (48)s > 4.41, *p*s < .001/6), in which the size of the largest chunks recalled by rated chess players was bigger for game positions (*M* = 4.56, *SD* = 1.67) than for random positions (*M* = 2.64, *SD* = 1.39), as well as bigger than non-chess players’ largest chunks for both game (*M* = 2.60, *SD* = 1.39) and random positions (*M* = 2.08, *SD* = 1.23). We inferred that rated chess players recalled bigger largest chunks for game positions while there was no difference between game and random positions for non-chess players. Besides, the significant main effect of age indicated that older players recalled smaller largest chunks.

When the analysis was restricted to rated chess players, we found all three main effects were significant (see Table 6 for results of the analysis). Rated chess players recalled bigger largest chunks for game positions than for random positions. Higher rated chess players recalled bigger largest chunks than lower rated players, and older rated chess players recalled smaller largest chunks. The interaction between chess skill and position type was not significant.

USCF ratings correlated significantly with the average size of the largest chunks only for game positions, but not for random positions (see Table 7).

### How do characteristics of repeated-recall chunks from recall of game positions relate to chess skill?

The traditional chunk characteristics of repeated-recall chunks were related to the amount of recall for game positions. In this section we examine two chess-related characteristics that were hypothesized to be associated with higher chess skill in the introduction. The first hypothesis was that the increased number of chess relations between consecutively recalled chess pieces within a chunk (see Chase and Simon [4, 25]) would be associated with a higher level of chess knowledge and skill. The second hypothesis was measured by the flexibility of recall order, namely whether the pieces within an identified chunk are recalled in the same or varied orders, when they are recalled on Trial 1 and Trail 2.

We had to restrict the analysis to the estimates of chunks produced in game positions, since chunks need to have a minimal size of at least 2–3 pieces. For the analysis of within-chunk chess relations at least 2 pieces per chunk was required, and for the analysis of the order of recall of chunks across two recall trials required at least 3 pieces to be successfully analyzed. It is worth noting there that two non-chess players contributed no data to the analysis of within-chunk chess relations and eight non-chess players contributed no data to the analysis of recall order of within-chunk pieces across trials, thus degrees of freedom in the following analyses were influenced correspondingly.

**Analysis of chess relations between pieces for within- and between-chunks.** We counted the number of different chess relations between 2 consecutively recalled pieces by applying Chase and Simon’s [4] technique, which distinguished five types of relations (same piece, same color, spatial proximity and attack/defense relations).

**Fig 5:**
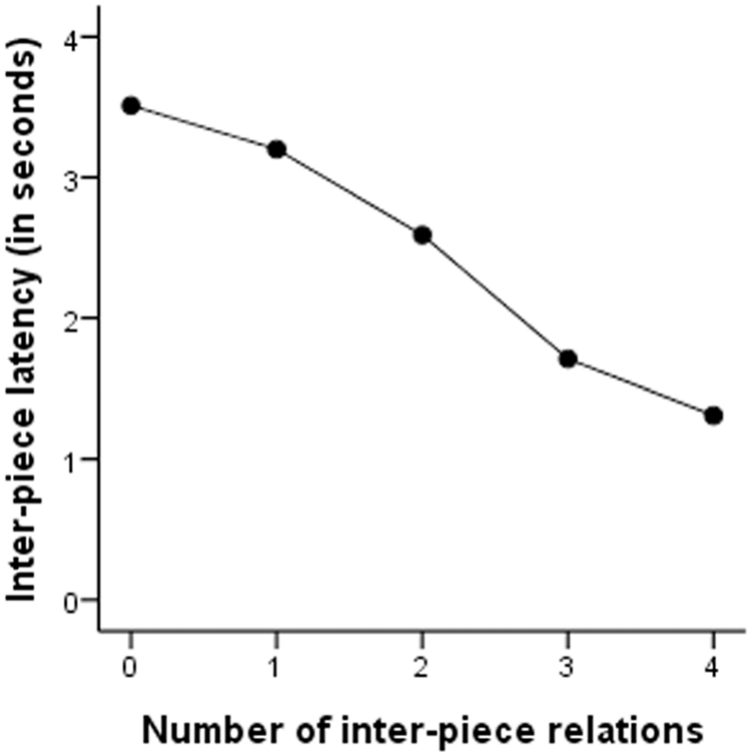
Inter-piece latency as a function of number of inter-piece chess relations (data pooled over type of positions and skill levels).

Consistent with Gobet and Simon [25, 4], the correlation between number of inter-piece chess relations and latencies was clearly negative, shorter latencies were associated with more chess relations. The function relationship between them can be seen in Fig. 5, where the data was pooled over all types of positions for all participants.

The question of particular interest concerning whether skill levels can be differentiated by an analysis of inter-piece relations. The average number of chess relations between consecutively recalled pieces in game positions was calculated for each participant separately for pieces within-a-chunk and pieces belonging to different chunks (between-chunk). This method eliminates the problem that certain players may recall many more pieces and thus would bias the analysis by contributing more data points. An LMM analysis of number of chess relations between two consecutively recalled pieces, by employing the main effects of age, skill group, and type of relation (within- versus between-chunk), the interaction between age and skill group, and the interaction between type of relation and skill group as fixed effects, showed that the only significant effect was found for the main effect of type of relation, *F* (1, 44.11) = 267.53, *p* < .001. The mean numbers of chess relations between 2 consecutively recalled pieces were 2.74 (*SD* = 0.34) for within-chunks and 1.18 (*SD* = 0.55) for between-chunks. In addition, no other effects were significant, all *F*s < 3.35, *ns*.

To better understand whether chess skill is related to different inter-piece chess relations, we excluded the superficial chess relations (same piece, same color and spatial proximity) and focused on the non-perceptual semantic relations, namely attack and defense. We counted the number of chunks which contained attack/defense relations and calculated the percentage of these chunks by divided total number of chunks (with at least 2 pieces contained) on each given position. A one-way ANOVA analysis showed a significant effect of skill group, *F* (1, 46) = 4.82, *p* < .05. The percentage of chunks which contained semantic relations was 62.78% (*SD* = .36) for chess players, and 36.23% (*SD* = .47) for non-chess players. We further calculated the average number of semantic relations within chunks and a one-way ANOVA analysis showed a significant main effect of skill group, *F* (1, 46) = 27.57, *p* < .001. Chess players recalled a significantly larger number of defense/attack relations (*M* = 0.47, *SD* = 0.16) than non-chess players (*M* = 0.23, *SD* = 0.17).

When the analysis was restricted to only the rated chess players, we found the average number of all chess relations within chunks significantly correlated with correct recall, but not with USCF rating. However, the average number of defense/attack relations did not correlate significantly with correct recall, nor with USCF rating (see Table 7).

**Fig 6:**
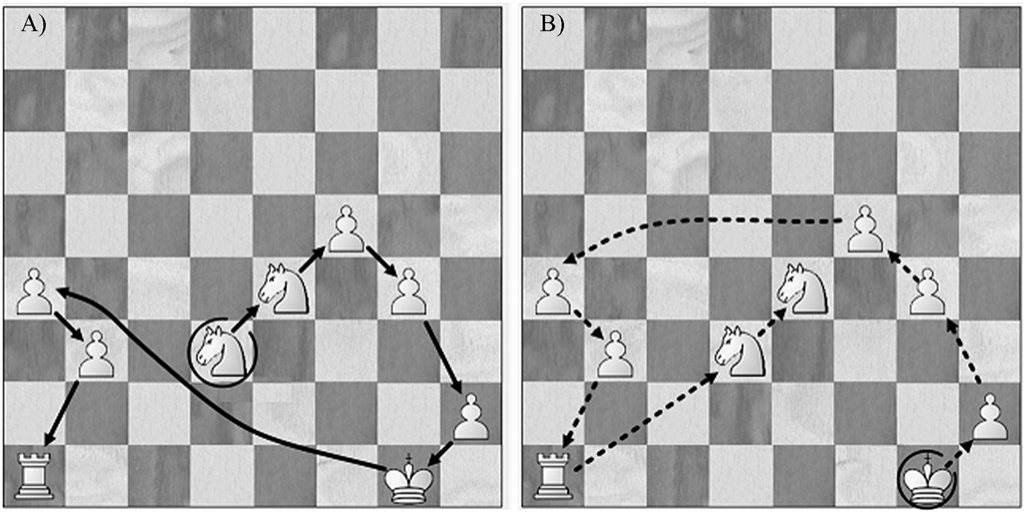
An example of a different ordered chunk recalled by a Class A chess player, where the open-circled piece indicates the start of recall: Arrows with full lines indicate the order of pieces recalled on Trial 1 (A), and arrows with dotted lines indicate the order of pieces recalled on Trial 2 (B).

**Analysis of order of recall of pieces within chunks.** Chunks consisting of three or more pieces could be recalled in three different types of orders during Trials 1 and 2. First, the chess pieces could be recalled in identical order on the two trials (Identical ordered chunk). Or the chess pieces could be recalled in the completely reverse order on the second trial (Reverse ordered chunk). Alternatively, the chess pieces could be recalled together in an order that was neither identical nor reverse on the second trial (Different ordered chunk). To illustrate the type of data analyzed we will give a couple of examples. As shown in Fig. 2, the participant recalled the castled king side in exactly the same orders on Trial 1 and Trial 2, which corresponded to an Identical ordered chunk. In contrast one of the participating Class A players recalled a complex Different ordered chunk across trials as is illustrated in Fig. 6. This large chunk could be viewed as consisting of three parts, and the orders of recall of the large chunk are not consistent across trials, even the orders of recalling the parts is different.

A one-way ANOVA analysis of percentage of Different ordered chunks using skill group as the independent variable showed that the main effect was significant, *F* (1, 40) = 32.98, *p* < .001, while the main effect of age (*F* < 1) was not significant. In sum, compared to non-chess players (*M* = 0.33, *SD* = 0.14), rated chess players (*M* = 0.82, *SD* = 0.41) were more likely to recall the within-chunk pieces in a more varied order on the two trials.

When we restricted the analysis to rated chess players, the correlations between percentage of Different ordered chunks, on the one hand, and number of pieces recalled correctly and USCF ratings, on the other, were not significant (see Table 7).

### Can characteristics of repeated-recall chunks predict chess skill among rated chess players?

Some characteristics of the identified repeated recall chunks were found to be significantly related to chess skill as measured by USCF ratings (see Table 7). A hierarchical multiple regression analysis was conducted using the four chunk characteristics derived from game positions and the two measures of recalled chess pieces as predictors of USCF rating. Once the sizes of the largest chunks were entered, *R* ^*2*^ = .22, *F* (1, 23) = 6.55, *p* < .05, the other characteristics did not account for any significant additional variance, *F*_change_ < 2.25, *ns*.

Consistent with our intuitions, when we calculated the correlations for the chunk characteristics derived from the recall of the random positions and USCF ratings, none of the correlations reached significance, *r* (23) < .30, *ns*.

## Summary and General Discussion

Our study was designed to contribute to the current debate about the role of chunks and their characteristics in the representation of chess positions by chess players. We were able to assess the effects of perceptual patterns independent of chess knowledge and skill by analyzing the recall of non-chess players, who were hypothesized to have acquired no previously acquired chunks related to chess playing. When we compared the recall and identified chunks of the non-chess players to those of rated chess players, we found an overall effect of chess skill in support of corresponding hypotheses based on chunking. At the same time our analyses of the chunks for the two types of participants revealed some interesting deviations from the assumptions of chunking theory. According to Chase and Simon [4] the differences between the number of relations of consecutive pieces within and between chunks reflected the structure of chess knowledge. In contrast we found no significant differences between non-chess players and chess players on the number of relations between consecutive pieces within chunks. This result is not consistent with Chase and Simon’s [4] hypothesis that the larger number of relations between consecutive pieces within chunks compared to between chunks provides evidence that chunks identified in chess players reflect the effects of playing and analyzing chess games. However, the differences in number of relations of pieces within chunks between non-chess players and rated players reached significance when we restricted the analysis to only semantic relations between consecutive chess pieces that concerned attack and defense relations. A more plausible hypothesis is that chess players’ memories of briefly presented game positions differ from that of non-chess players because their memory includes semantic chess information. However, the frequency of these relations is low compared to more perceptually salient relations, such as same color or same piece, encoded by all participants and thus the overall number of relations failed to reach statistical significance. On the other hand, the difference between rated players and non-chess players in their reliance on conceptual chess relations is consistent with chunks providing information for move selection as argued by chunking theory.

More generally our analyses of chunks of non-chess players and rated chess players showed large differences consistent with the differences in chess knowledge and skill. We found large differences but only for chunks from game positions, not from random positions. The traditional physical characteristics of chunks, namely size of largest chunk and number of chunks (see Table 7) showed a large superiority for the rated chess players. The pattern of significant findings between rated players and non-chess players in Table 7 is consistent with the rated chess players’ knowledge of chess compared to no such knowledge among the non-chess players.

To assess the relevance for chess skill, we examined the correlations between the USCF ratings of the rated chess players and chunk characteristics assessed from the recall of game positions. The size of the largest chunk recalled in game positions correlated significantly. This significant relation is consistent with chunking theory’s predictions as it relates to acquisition of bigger chunks. Although memory performance for game positions was correlated with several different chunk characteristics, the size of the largest chunk singularly accounted for all the significant covariance between chess skill and all the other three characteristics among rated players.

In sum, the correlation between memory performance and USCF rating is well established. However, our analysis was unable to identify any significant mediators beyond the size of the largest chunk. Further research with increased statistical power is needed to describe how the amount of recall of chess pieces and larger chunks are related in greater detail to the probability of recall of particularly relevant chess relations for the next move selection for the presented chess position. Besides, consistent with Charness [34], we identified one finding that is suggestive of such independence, namely the age effect on the size of chunks, where older rated players recalled smaller chunks without any significant interaction with USCF ratings.

### The benefits of using several different techniques for identifying chunks

The primary finding was that two very different techniques (RT technique and. repeated-recall technique) identified closely related chunks with similar characteristics. When we conducted a direct comparison of the reliability and validity of assessed chunk characteristics, we found a significant advantage for the repeated-recall technique. One possible reason for this superiority is that the repeated-recall technique does not differentiate between traditional pre-existing chunks [3, 4], templates [8], hierarchical chunks [28, 29] or memory encodings in Long-Term Working Memory formed at the time of the original presentation of the chess position [40–43]. It seeks to identify independent structures in LTM without seeking to assess their internal structure or whether these structures had been previously acquired in LTM prior to the encounter during the initial presentation of the stimulus. Consistent with earlier research on chunks identified with the RT technique, we found that the repeated-recall technique identified chunks where the number of relations between chess pieces recalled consecutively within a chunk were larger than between chess pieces recalled consecutively but belonging to two different chunks.

The repeated-recall technique allows us to examine aspects of chunks that cannot be studied with a single recall with the RT technique. It is possible to study order of recall of chess pieces within chunks and this should allow researchers to describe a more detailed structure of the recalled chess pieces. Our analysis of recall order showed that rated players were more varied in their order of recall of chess pieces within chunks than non-chess players. By using the RT technique and the repeated recall technique in combination it should be possible to distinguish different types of more complex structures in the chess players’ recall. We would recommend that future researchers conduct a more systematic analysis of successive multiple recall trials to identify how players are able to add information about the structure of the chess position across trials. Such a detailed analysis should be able to identify differences in the structure of recall that would allow discrimination between pre-existing chunks and templates [8], on the one hand, and other proposals for how the chess positions are encoded in LTM at the time of the initial presentations and after subsequent repeated presentations to eventually allow the participant to produce a perfect reproduction of the chess position.

### Limitations and suggestions for further research

The failure to uncover significant relations between characteristics of chunks and chess skill among the chess players may always be an issue of statistical power. Our failure to identify characteristics of recall of game positions that explained significant variance in chess skill beyond the size of the largest chunks may be due to the small number of chess positions presented in each condition. In the current study we only presented two chess positions in each experimental condition. However, other studies relating recall of chess positions to chess skill have used either two [13] or three positions [33] in each experimental condition. By focusing on studying rated chess players and constraining trials to recall of game positions presented at a single presentation time (5 s), future studies would be able to use two repetitions of presentation and recall (c.f. [22]) and thus include a larger number of positions and have greater statistical power to find significant relations between the new characteristics and chess skill.

Another limitation concerns the relation between memory performance and chess skill and the relatively low ratings of the studied chess players. We succeeded in recruiting chess players from a wide range of skill levels, but there were only a few highly skilled players in the current study and we were not able to recruit any chess masters. A future study needs to recruit more highly skilled chess players so the range of chess skill would be more completely covered to increase the likelihood of finding characteristics of chunks that would be highly correlated with both memory performance as well as chess skill.

With respect to the repeated-recall technique itself, we admit that (parts of) chunks could be encoded in memory after the first cycle of position presentation and reconstruction. Finally, if one is interested in studying the memory representations that mediate individual differences in chess skill and, in particular, superior move selection, it may not be optimal to study memory performance directly. It might be more appropriate to study the selection of the best move for a presented game position and then test for memory in a manner similar to the one used by de Groot [44] in his original studies [41, 45]. This method, focusing on expert performance in chess, is likely to identify the patterns of chess pieces that are considered during the generation of better moves by more skilled players. During trials with this method one can make strong predictions about the relation of recalled chess pieces and the selected move. It is possible that the characteristics of chunks recalled after move-selection trials might differ from trials with the explicit task of recalling as many pieces as possible from the presented position.

### Theoretical implications

The primary goal of our article is to address the recent debate about whether or not chunks identified from recall of chess positions can differentiate higher skilled players from lower skilled players [9, 10]. As summarized above, with the exception of the perceptual relations between consecutively recalled chess pieces within chunks, our analysis showed that non-chess players differed significantly from chess players for all characteristics of chunks in game positions. We can, therefore, conclude that higher skilled chess players can be differentiated from lower skilled players by the structure and content of chunks.

Our finding that more highly rated chess players encoded more abstract and semantic chess relations seems to qualify Linhares and Freitas’ [9] criticisms of chunking theory and their remarks that chunks contained very little chess related information and could not discriminate between different levels of chess knowledge. We believe that it is very important to recognize that the work on recalled chunks is based on a different paradigm than the work cited by Linhares and his colleagues [22]. Although both lines of research on memory for chess positions have presented game positions for 5 s, Linhares et al. [22] allowed their chess players two cycles of presentation and recall. Most of the rated chess players studied by Linhares et al. [22] were notably superior in skill to those in our current study. We interpret our findings to show the ability of better chess players to encode semantic information at a lower degree than the more complete and advanced encodings of the higher skilled players with two available attempts to see and reproduce the positions. In addition, the analyses of chess relations also indirectly suggest that skilled chess players construct chess positions based on both basic elements and semantic abstractions, while non-chess players mainly rely on basic elements.

Future research should be encouraged to study individual participants’ encoding methods by collecting retrospective recall protocols and designing experiments to test hypotheses about the detailed memory skills. Several studies of this type have successfully described the detailed structure of long-term working memory for many memory experts [42, 43, 46–49]. In chess, one of the most important contributions to research on chess memory was conducted by Gobet and Simon [8], who studied Gobet’s memory performance for multiple chess positions for many months. If we were able to obtain data from several other individual participants with the addition of verbal protocols, then our understanding of chess skill is likely to increase considerably.

## Supporting Information

S1 File Raw data file. (XLSX)
